# Differences in the amplitude of low-frequency fluctuations of spontaneous brain activity between preterm and term infants

**DOI:** 10.3389/fneur.2024.1346632

**Published:** 2024-03-01

**Authors:** Ye Feng, Yuanchong Wang, Xu Li, Liying Dai, Jian Zhang

**Affiliations:** ^1^Department of Neonatology, Anhui Provincial Children’s Hospital, Hefei, China; ^2^Department of Pediatric Medicine, Anhui Provincial Children’s Hospital, Hefei, China; ^3^Department of Imaging, Anhui Provincial Children’s Hospital, Hefei, China; ^4^Neonate Follow-up Center, Anhui Provincial Children’s Hospital, Hefei, China

**Keywords:** resting-state functional magnetic resonance imaging, the amplitude of low-frequency fluctuation, preterm infants, term infants, spontaneous activity

## Abstract

**Objectives:**

To date, the majority of research on resting-state functional magnetic resonance imaging (rs-fMRI) in the developing brain has primarily centered on adolescents and adults, leaving a gap in understanding variations in spontaneous brain activity at rest in preterm infants. This study aimed to uncover and comprehend the distinctions in spontaneous brain activity between preterm and term infants, with the goal of establishing a foundation for assessing the condition of preterm infants.

**Methods:**

In this study, 14 term infants and 15 preterm infants with equivalent gestational age were carefully chosen from the neonatal unit of Anhui Provincial Children’s Hospital. The amplitude of low-frequency fluctuations (ALFF) intensity was assessed using resting-state functional magnetic resonance imaging (rs-fMRI) to examine brain activity in both groups. Subsequently, the differences between the term and preterm infants were statistically analyzed using a two-sample *t*-test. A *p-*value of <0.05, corrected for the REST Gaussian Random Fields, was deemed to be statistically significant.

**Results:**

In comparison to the term infant group, the preterm infant group exhibited a significant increase in the ALFF value in the left precuneus, left frontal superior orbital gyrus, and left calcarine cortex.

**Conclusion:**

Significant variances in spontaneous brain activity have been observed in various regions between term infants and preterm infants of equivalent gestational age. These variations could potentially impact the emotional and cognitive development of preterm infants in the long term.

## 1 Introduction

The World Health Organization defines preterm infants as newborns born at less than 37 weeks of gestational age (1). Globally, approximately 15 million preterm infants are born each year, representing a significant proportion of the population (2). While the survival rate of preterm infants has improved significantly with the development of perinatal medicine and advancements in neonatal care technology, the long-term neurodevelopmental outcomes for these infants have not followed suit, making preterm birth the primary cause of neurological disability in children. Both high-risk preterm infants (gestational age < 34 weeks) and low-risk preterm infants (gestational age ≥ 34 weeks) have higher rates of developmental delay and neurodevelopmental abnormalities compared with term infants (3). Furthermore, the younger the gestational age, the higher the risk of neurodevelopmental disorders and the greater the likelihood of requiring school-age support (4). Previous research has primarily utilized conventional magnetic resonance imaging or diffusion-weighted magnetic resonance imaging to identify brain structural abnormalities in preterm infants (5). However, some research has shown that preterm infants with significant structural brain damage not detected by conventional magnetic resonance imaging may also experience cognitive impairment and behavioral abnormalities during childhood (6). This suggests that studying neonatal brain development and brain injury at the functional level is more accurate and sensitive.

Since the 1990s, functional imaging technologies such as functional magnetic resonance imaging, positron emission tomography, single photon emission computed tomography, magnetoencephalography, and optogenetic functional magnetic resonance imaging have undergone rapid advancements. These technologies have significantly contributed to the field of brain function research, disease diagnosis, and treatment. Each of these tests possesses its own advantages, but they also have certain limitations. Among them, resting-state functional magnetic resonance imaging (rs-fMRI) is a recent innovation in magnetic resonance imaging that does not require specific tasks and offers significant advantages in the investigation of neonatal brain function. Rs-fMRI has the ability to enhance prognostic assessments of brain injury by detecting the extent of damage in various brain functional regions, offering excellent temporal and spatial resolution. Using rs-fMRI, it is possible to identify the resting network, a large functional network that exhibits coordinated fluctuations in blood oxygen level-dependent (BOLD) signals linked to spontaneous brain activity in infants (7, 8). Currently, rs-fMRI has been used to study functional phylogeny in children (9) and to assess functional system integrity in adults (10). Prior research conducted on older children and adults has also identified rs-fMRI abnormalities in preterm infants aged 12 to 14 years (11–13). Therefore, rs-fMRI provides a tool to study functional brain development abnormalities caused by preterm birth.

Previous studies have found that both term infants and preterm infants exhibit coherent low-frequency fluctuating BOLD signals in a wide range of functionally relevant brain regions (14, 15). The amplitude of low-frequency fluctuation (ALFF) is commonly used to reflect spontaneous brain activity by measuring spontaneous low-frequency oscillations of the BOLD signal for each voxel in the brain without any predefined seed region of interest (16). Currently, there is extensive research on various psychiatric disorders using ALFF, including attention deficit hyperactivity disorder (17), autism spectrum disorder (18), and Parkinson’s disease (19). There is also evidence suggesting that ALFF may serve as a novel biomarker for the physiological state of the brain. Therefore, we employed the ALFF approach to evaluate the differences in spontaneous brain activity between preterm and term infants at rest.

The aim of this study is to assess the intensity of spontaneous activity in resting brain neurons using the ALFF algorithm through simultaneous rs-fMRI of term infants and preterm infants of equivalent gestational age. Subsequently, the ALFF values of the preterm infant group and the term infant group were compared to analyze the differences in the spontaneous activity of brain neurons. This approach allows us to not only map the trajectory of normal brain development during this period, but also understand the impact of preterm birth on brain development.

## 2 Materials and methods

### 2.1 Participants

We enrolled 71 newborns (born between April 2021 and February 2022) from the neonatal unit of Anhui Provincial Children’s Hospital, comprising 36 preterm infants of equivalent gestational age (28^+5^–36^+6^ weeks gestation; birth weight between 900 g and 2,750 g) and 35 term infants (37–42 weeks gestation; birth weight between 2,550–4,750 g). This study was approved by the ethics committee of the Anhui Provincial Children’s Hospital. Parental informed consent was obtained from each subject prior to cranial magnetic resonance examination.

Anatomical magnetic resonance imaging (MRI) for all subjects was reviewed by a radiologist and a pediatrician. Children with grade II or greater intraventricular hemorrhage, inherited metabolic encephalopathies, hypoglycemic encephalopathies, bilirubin encephalopathies, and congenital brain developmental abnormalities were excluded from the study. The establishment of these inclusion criteria provides us with a suitable study population for our research and helps minimize potential confounding factors.

### 2.2 Data acquisition

Term infants underwent MRI approximately 10 days after birth, while preterm infants underwent MRI at around 37 to 40 weeks of postmenstrual age. Both groups of infants were imaged under sedation (midazolam: <2 mg/kg). Earmuffs were utilized to minimize noise during the scanning process, and a neonatal nurse continuously monitored the arterial oxygen saturation and heart rate of the infants throughout the acquisition process, halting the image acquisition immediately in the event of an unforeseen event.

The MRI scan was performed in the MRI room of the Department of Imaging, Anhui Provincial Children’s Hospital. Imaging was performed using a 1.5 T Philips Achieva fMRI (Royal Philips Electronics, Netherlands). Structural images were acquired using T1W-3D-TFE image-weighted columns (parameters: TR 0.0074 s, TE 0.0034 s, flip angle 8°, acquisition time 178.1 s, field of view 250 mm × 259 mm, matrix 228 × 227, number of layers 180). Acquisition of FE-EPI data using gradient echo imaging sequences (TR 2.5 s, TE 0.05 s, flip angle 90°, field of view 200 mm × 175 mm, matrix 64 × 53, layer thickness 3.75 mm, number of layers 28, Acquisition time 382.5 s).

### 2.3 Data preprocessing

The RESTplus v1.28^1^ data analysis toolkits were used to pre-process and analyze the images. The preprocessing procedure involved several steps: (1) Conversion of DICOM-format data to NIFITI-format data; (2) Removal of the first 10 time points to reduce the instability of the magnetic field at the beginning of the scan; (3) Slice timing correction to ensure accurate representation of the temporal order of brain activity; (4) Head movement correction and removal of data with large head movements (maximum translation >3 mm or maximum rotation >3° in all three directions) to ensure data accuracy and reliability for subsequent analysis; (5) Space standardization involving linear registration to roughly match the individual neonatal brain structure with the standard template, followed by non-linear registration to further refine the alignment of the neonatal brain imaging data with the neonatal template (20) to eliminate individual differences, making the data more comparable and consistent; (6) Spatial smoothing using a Gaussian kernel with a full width at half maximum (FWHM) of 6 mm prior to ALFF calculation to reduce noise in the data and enhance the signal-to-noise ratio; (7) Removal of linear trend to allow for a clearer analysis of the underlying patterns or variations; (8) Nuisance covariates regression involving the regression of eight signals, including the six parameters obtained through head motion correction and the white matter cerebrospinal fluid to isolate the specific effect of the independent variable on the dependent variable. Finally, each subject’s data was manually checked for registration errors and signal loss. Following these exclusion criteria, 42 participants were excluded from the dataset, leaving 15 preterm and 14 term infants for inclusion in the follow-up analysis.

## 3 ALFF analysis

RESTplus v1.28 (see Footnote 1) was utilized to calculate the ALFF values. Following data preprocessing, the ALFF values were computed using the following steps: fast Fourier transform was employed to convert each voxel’s time series to the frequency domain, and the power spectrum for each voxel was computed. The ALFF values were then derived by averaging the square root of each frequency of the power spectrum at each voxel over the 0.01 to 0.08 Hz range (13). Subsequently, the raw ALFF values were converted to Z-scores for use in group comparisons.

## 4 Statistical analysis

The descriptive statistical analysis of general subject demographics involved calculating measurement data in terms of mean values and standard deviations, and representing count data in terms of the number of cases and frequency. Chi-squared tests and independent samples *t*-tests were performed using SPSS 23.0 (SPSS Inc., USA) with a statistical significance level of *p* < 0.05.

Differences in ALFF between the two groups were calculated using a two-sample *t*-test based on the Data Analysis Toolkit SPM12,^2^ When conducting a two-sample *t*-test, we used the neonatal template (20) as a mask and after correcting for REST Gaussian random field, *p* < 0.05 was considered statistically significant.

## 5 Results

### 5.1 Participants demographics

There were no significant differences in gender, hypertension in pregnancy, gestational diabetes mellitus, prenatal magnesium sulfate therapy between term infants and preterm infants (*p* > 0.05). However, significant differences were noted in gestational age at birth, birth weight, mode of delivery, and prenatal hormone treatment between the two groups (*p* < 0.05) as shown in Table 1.

**Table 1:** Demographic and clinical features of the preterm infants and term infants.

Category	Preterm newborns (*n* = 15)	Term newborns (*n* = 14)	*t/*χ^2^	*P*
Gestational age (mean ± SD weeks)	33.21 ± 2.74	39.00 ± 1.34	−7.143	<0.01
Birthweight, (mean ± SD g)	2066.00 ± 790.59	3258.75 ± 727.98	−4.217	<0.01
Male, *n* (%)	8 (53%)	11 (79%)	2.042	>0.05
Vaginal delivery, *n* (%)	6 (40%)	12 (86%)	6.427	<0.05
Hypertension in pregnancy, *n* (%)	3 (20%)	3 (21%)	0.009	>0.05
Gestational diabetes mellitus, *n* (%)	1 (7%)	0	0.967	>0.05
Prenatal magnesium sulfate therapy, *n* (%)	3 (20%)	0	3.123	>0.05
Prenatal Hormone treatment, *n* (%)	4 (27%)	0	4.331	<0.05

### 5.2 Aberrant local activity

**Figure 1:**
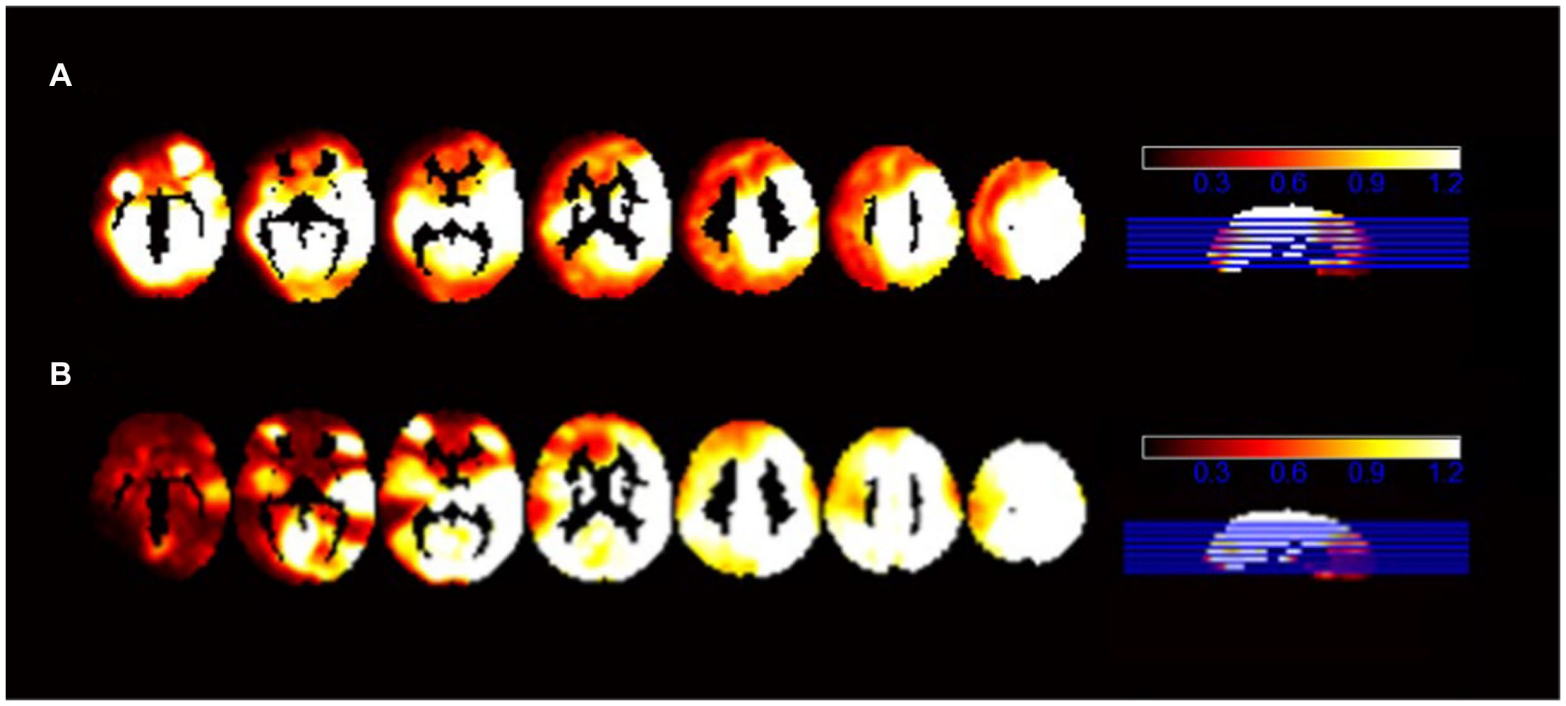
The ALFF brain maps of individual term infants and preterm infants. **(A)** Represents the ALFF brain map of term infants, and **(B)** represents the ALFF brain map of preterm infants.

**Figure 2:**
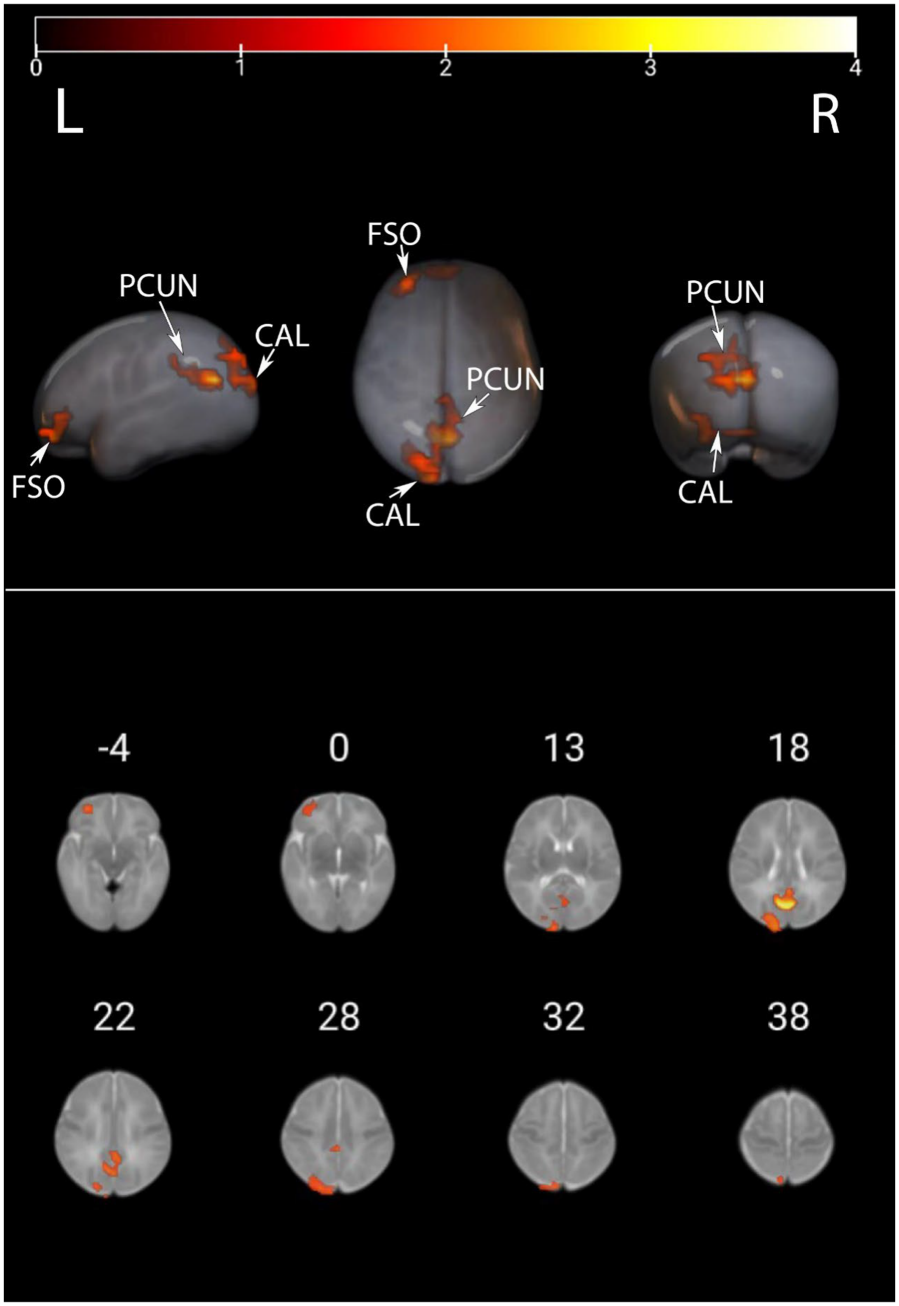
ALFF values were compared between preterm infants and term infants. This image displays the differences in ALFF values between preterm and term infants. Warmer colors represent elevated areas. These include the left precuneus, left frontal superior orbital gyrus, and left calcarine cortex as described in further detail in Table 2. PCUN, precuneus; CAL, calcarine cortex; FSO, frontal superior orbital gyrus.

We presented the ALFF brain maps of individual term infants and preterm infants in Figure 1, where a represents term infants and b represents preterm infants. The ALFF brain map of the preterm group was subtracted from the term group to obtain the contrast image. We found abnormal changes in several regions of the brain in preterm infants (Figure 2). The results showed that the preterm infant group had higher ALFF in the left precuneus, left frontal superior orbital gyrus, and left calcarine cortex than the term infant group (Table 2).

**Table 2:** Comparison of ALFF values in different brain regions between preterm infants and term infants.

Brain region	BA	voxel	MNI (x, y, z)	*t-*value
Left precuneus	29	88	−5, −48, 18	3.95
Left calcarine	17	113	−8, −68, 14	2.45
Left frontal sup orb	11	68	−22, 32, −12	2.73

BA, Brodmann area; MNI (x,y,z), coordinates in the Montreal Neurological Institute space; *t* value, two-sample *t* tests of the peak voxel (showing the largest significant difference within a cluster).

## 6 Discussion

Utilizing rs-fMRI, the analysis of ALFF values revealed variations in spontaneous brain activity among preterm infants with equivalent gestational age. Significant disparities in ALFF were identified in the left precuneus, left frontal superior orbital gyrus, and left calcarine cortex between the two groups. Notably, higher ALFF values were detected in the left precuneus, left frontal superior orbital gyrus, and left calcarine cortex within the preterm infant group when compared to the term infant group.

The precuneus, located in the medial hemisphere of the brain, is a part of the parietal lobe and is associated with numerous higher cognitive functions, including visuospatial abilities, mental imagery, episodic memory, emotional processing and mentalization (21). Extensive research has explored the role of the precuneus in various psychiatric diseases, such as schizophrenia (22), Huntington’s disease (23), chronic depression (24), Parkinson’s disease (19), Alzheimer’s disease (25), childhood autism (26–28), and others. It is hypothesized that abnormal activity of the precuneus in early preterm infants may be linked to their subsequent vulnerability to psychiatric disorders. Additionally, the precuneus is associated with episodic memory and has been shown to be involved in implicit memory in newborns, including preterm and term infants (29, 30). For example, perinatal maternal sounds (31–34) can be memorized by fetuses and newborns, including preterm infants and term infants. Most preterm infants require admission to the neonatal intensive care unit after birth, and exposure to various noxious stimuli in the neonatal intensive care unit setting can lead to the formation of implicit stimuli memory in these preterm infants (35), which can also adversely affect brain structure and function in the early years (36, 37). In contrast, term infants rarely encounter such stimuli in their home environment, which may account for the stronger activity of the precuneus observed in early preterm infants compared to term infants. The precuneus is also recognized as a visual area, situated between the visual and sensorimotor areas. However, years of clinical experience have demonstrated that surgical invasion of the precuneus, particularly if the occipital junction remains intact, does not uniformly result in significant visuospatial processing or visual integration defects (38–40). However, a review of the literature did not reveal any studies that linked unilateral precuneus dysfunction in preterm infants to visual acuity in later stages. Therefore, we hypothesized that the impact of the precuneus on visual acuity may be minimal or that bilateral precuneus dysfunction would have a more significant impact on vision.

The calcarine cortex, a sensory granular cortex located on the medial side of the occipital lobe, is primarily responsible for the processing of visual information (41). Marine et al. (42) observed an increase in the number of functional network connections in visual regions in very preterm infants of equivalent gestation compared with healthy controls, aligning with the findings of Padilla et al. (43). This may be attributed to the significant increase in synaptic density in the visual cortex from the second trimester to the first few months of life. Early extrauterine exposure may lead to an increase in visual stimuli received by preterm infants, ultimately leading to increased activity in the calcarine cortex (44–46). We speculate that the increase in visual information input during this critical period may have an impact on the activity of the visual area. However, there is currently no evidence to suggest a relationship between the enhancement of visual regional activity in early preterm infants and their subsequent visual processing impairments. In addition, increased ALFF values were also detected in the left frontal superior orbital gyrus. The frontal superior orbital gyrus is situated near the orbitofrontal region, which is close to the nasal cavity and sinuses. It contains a significant amount of gas, which can easily lead to image distortion due to the uneven static magnetic field, especially in echo plane imaging (47). To address this issue, you can utilize the distortion correction method known as MEDIC (Multi-Echo Distortion Correction) to accurately estimate the B0-related distortion of each frame of multi-echo fMRI data (48). In comparison to the previous gold-standard method, i.e., TOPUP, MEDIC’s frame distortion correction enhances anatomical alignment and mitigates the impact of head motion on resting-state functional connectivity diagrams in datasets with higher motion (48, 49). However, it requires obtaining multiple echoes during a single-plane EPI readout, which we are currently unable to address, but we aim to improve this in our future studies.

There have been few previous studies on ALFF values in different brain regions of preterm infants. When Wu et al. (50) examined ALFF values in late preterm infants, they observed increased activity in brain regions such as the posterior cingulate/precuneus gyrus, infratemporal gyrus, and orbitofrontal cortex when compared to term infants during the same period. In preterm infants, ALFF activity is reduced in the primary somatosensory cortex, the precentral gyrus, the superior temporal gyrus, the calcarine cortex, the cuneus, and the superior occipital gyrus. However, our studies did not detect differences in other brain regions, with the exception of the precuneus and calcarine. This is presumably due to the small sample size. Therefore, it is necessary to enroll a larger number of children in future studies aimed at further characterizing the characteristics of spontaneous brain activity in preterm infants. This will aid in our understanding of the neural mechanisms underlying prematurity and enable us to provide timely and targeted interventions.

## 7 Main limitations and future perspectives

A limitation of this study is the small sample size, which does not accurately represent the abnormalities of early brain function development in all individual preterm infants. To enhance the accuracy of clinical assessment, the newborns were mildly sedated with midazolam, which may have influenced their brain activity and thus influenced the test results. Therefore, future studies should aim to expand the sample size and image neonates in their natural sleep state.

Another limitation of this prospective study is the absence of long-term follow-up and the exclusive reliance on functional magnetic resonance imaging to assess brain development in preterm infants. Therefore, future efforts should also prioritize longitudinal assessment of preterm infants throughout early childhood and incorporate other complementary modalities to comprehensively evaluate brain development and function, such as functional near-infrared spectroscopy, electroencephalogram, diffuse reflection optical tomography, behavioral development scales, and more. Despite their limitations, these methods are becoming increasingly accepted and are utilized at most institutions.

## 8 Conclusion

Using rs-fMRI data, the preterm infant group exhibited higher ALFF values in the left precuneus, left frontal superior orbital gyrus, and left calcarine cortex compared to the term infant group. The findings of this study suggest that there are distinct differences in spontaneous brain activities between term and preterm infants of equivalent gestational age in various brain regions. This disparity may have adverse effects on the long-term mood and cognitive development of preterm infants.

## Data availability statement

The original contributions presented in the study are included in the article/supplementary material, further inquiries can be directed to the corresponding author.

## Ethics statement

The studies involving humans were approved by Ethics committee of the Anhui Provincial Children’s Hospital. The studies were conducted in accordance with the local legislation and institutional requirements. Written informed consent for participation in this study was provided by the participants' legal guardians/next of kin. Written informed consent was obtained from the minor(s)' legal guardian/next of kin for the publication of any potentially identifiable images or data included in this article.

## Author contributions

YF: Data curation, Writing – original draft. YW: Data curation, Investigation, Writing – original draft. XL: Data curation, Writing – original draft. LD: Writing – review & editing. JZ: Conceptualization, Methodology, Writing – review & editing.

## Footnotes

^1^ http://www.restfmri.net/forum/

^2^ https://www.fil.ion.ucl.ac.uk/spm/software/spm12/
